# Intra- and Interhemispheric Propagation of Electrophysiological Synchronous Activity and Its Modulation by Serotonin in the Cingulate Cortex of Juvenile Mice

**DOI:** 10.1371/journal.pone.0150092

**Published:** 2016-03-01

**Authors:** Víctor Rovira, Emilio Geijo-Barrientos

**Affiliations:** Instituto de Neurociencias, Universidad Miguel Hernández–CSIC, San Juan de Alicante, Alicante, Spain; University of Salamanca- Institute for Neuroscience of Castille and Leon and Medical School, SPAIN

## Abstract

Disinhibition of the cortex (e.g., by GABA -receptor blockade) generates synchronous and oscillatory electrophysiological activity that propagates along the cortex. We have studied, in brain slices of the cingulate cortex of mice (postnatal age 14–20 days), the propagation along layer 2/3 as well as the interhemispheric propagation through the corpus callosum of synchronous discharges recorded extracellularly and evoked in the presence of 10 μM bicuculline by electrical stimulation of layer 1. The latency of the responses obtained at the same distance from the stimulus electrode was longer in anterior cingulate cortex (ACC: 39.53 ± 2.83 ms, n = 7) than in retrosplenial cortex slices (RSC: 21.99 ± 2.75 ms, n = 5; p<0.05), which is equivalent to a lower propagation velocity in the dorso-ventral direction in ACC than in RSC slices (43.0 mm/s vs 72.9 mm/s). We studied the modulation of this propagation by serotonin. Serotonin significantly increased the latency of the intracortical synchronous discharges (18.9% in the ipsilateral hemisphere and 40.2% in the contralateral hemisphere), and also increased the interhemispheric propagation time by 86.4%. These actions of serotonin were mimicked by the activation of either 5-HT_1B_ or 5-HT_2A_ receptors, but not by the activation of the 5-HT_1A_ subtype. These findings provide further knowledge about the propagation of synchronic electrical activity in the cerebral cortex, including its modulation by serotonin, and suggest the presence of deep differences between the ACC and RSC in the structure of the local cortical microcircuits underlying the propagation of synchronous discharges.

## Introduction

The cingulate cortex in the rat is defined by Vogt and Peters [1] as the portion of the cortex on the medial surface of the hemisphere that surrounds the callosal sulcus. This cortical region is similar in mice and consists of two distinct areas in terms of cytoarchitectonics, connections, and function: the anterior cingulate cortex (ACC) and the retrosplenial cortex (RSC) [1, 2]. The ACC can be subdivided further in dorsal (area 24b) and ventral (area 24a) agranular areas [3]; the RSC, which occupies the caudal half of the cingulate cortex, is divided in a dorsal agranular area (or dysgranular, due to the presence of a rudimentary layer 4 [4]; area 29d [1] or 30 [2]) and a ventral granular area (areas 29a–c) in the rat and mouse [1, 2]. The granular (ventral) RSC is characterized by an enhanced layer 2 formed by densely packed, callosal projecting small pyramidal neurons whose dendrites form tight bundles in layer 1 [1, 5, 6]; these superficial small pyramidal neurons show a characteristic late-spiking firing pattern [7]. The ACC and RSC are densely interconnected [8, 9, 10, 11] and are implicated in a wide range of cognitive functions, including sensory processing (particularly pain processing), memory processing, spatial learning, and navigation [12, 13, 14, 15].

A functional property of the cerebral cortex is the generation of synchronized activity during normal sensory processing [16] and also during pathological conditions (e.g., seizures [17]). Disinhibition of the neocortex by blockers of GABA_A_ receptors (bicuculline, picrotoxin) is especially effective in generating synchronous activity, both *in vivo* [18] and *in vitro* [19, 20, 21]. Although there are studies about the mechanism involving the generation and local propagation of this synchronized activity in motor cortical areas [18, 22, 23], including the propagation of epileptiform discharges across the corpus callosum [24, 25], there is still little information about the propagation of this kind of electrical activity in other cortical areas.

Among other afferent systems, the cerebral cortex receives serotonergic fibers originating predominantly from the dorsal and, to a lesser extent, the median raphe nuclei [26, 27, 28]. This innervation is widespread and, at least in rodents, exhibits only moderate variations in terms of areal and laminar density [29]. Serotonin (5-HT) plays an important role in the modulation of cortical electrical activity [30] and 5-HT acts on cortical neurons, which express a variety of receptors for 5-HT, particularly the 5-HT_1A_, 5-HT_1B_, and 5-HT_2A_ receptor subtypes [31, 32, 33, 34, 35]. The 5-HT_1B_ receptor is expressed mostly in presynaptic terminals, and the 5-HT_1A_ and 5-HT_2A_ subtypes are expressed in pyramidal and non-pyramidal neurons, with a high degree of co-expression of these latter two subtypes in pyramidal neurons [36]. The actions of 5-HT on cortical neurons are complex; the pyramidal neurons of layer 5 of the rat neocortex are hyperpolarized or depolarized by 5-HT, with most cells able to show both responses [37, 38, 39]. On synaptic transmission, 5-HT exerts presynaptic and postsynaptic neuromodulatory actions in various types of neurons. In the presynaptic terminal, 5-HT mostly inhibits the release of transmitters [39, 40, 41, 42], thereby modulating synaptic efficacy. In the cerebral cortex, these effects are mediated by 5-HT_1B_ [39, 42, 43] and 5-HT_2A_ [43] receptors. Particularly important is to note the antiepileptic effect of 5-HT [44, 45], as well as the fact that the 5-HT receptor subtypes 5-HT_1A_ and 5-HT_2A_ are the targets of atypical antipsychotic drugs [46]. These drugs, which in the recent years have been substituting the use of classical antipsychotics, have an effect of blockade of 5-HT_2A_ receptors and activation of 5-HT_1A_ receptors, while they do not have an effect on dopamine receptors (and therefore, they do not produce extrapyramidal side effects, which are the main limitation of typical antipsychotic drugs).

In this work, we have studied the propagation of synchronous electrical activity evoked after the blockade of GABA_A_ receptors along layer 2/3 of both the ACC and RSC of the mouse. The generation and propagation of synchronous discharges along the cortex are closely related to the pathophysiology of epilepsy [47] and therefore, detailed knowledge of these phenomena will be useful for better knowledge of the electrophysiological mechanisms underlying epilepsy. We have also studied the role of the corpus callosum in the propagation of this activity to the contralateral hemisphere and the modulation by 5-HT of this intra- and interhemispheric propagation.

## Materials and Methods

### Ethical approval

Mice were maintained, handled, and killed in accordance with national and international laws and policies (Spanish Directive “Real Decreto 1201/2005”; European Community Council Directive 86/609/EEC). The Ethical Committee for Experimental Research of the Universidad Miguel Hernández approved the experimental protocols used.

### Slice preparation and recordings

Cortical slices of 400 μm thickness were prepared from male C57BL/6 mice (postnatal days 14 to 20) according to routine methods used in our laboratory [43]. Animals were killed by cervical dislocation, and coronal slices, in which a large part of the axons forming the corpus callosum remained intact, were cut with a vibratome (Leica VT1000) in an ice-cold cutting solution (composition in mM: NaCl, 124; KCl, 2.5; PO_4_H_2_Na, 1.25; Mg Cl_2_, 2.5; Ca Cl_2_, 0.5; NaCO_3_H, 26; glucose, 10; pH 7.4 when saturated with 95% O_2_ and 5% CO_2_). The slices were transferred to modified ACSF (composition in mM: NaCl, 124; KCl, 5; PO_4_H_2_Na, 1.25; Mg Cl_2_, 1; Ca Cl_2_, 1.2; NaCO_3_H, 26; glucose, 10; pH 7.4 when saturated with 95% O_2_ and 5% CO_2_), where they were incubated at 37°C during 30 min and thereafter kept at room temperature until use.

For recording, the slices were placed in a submersion type chamber and superfused at a flow rate of 3–5 ml/min with modified ACSF at 33–34°C. Extracellular recordings were obtained with glass microelectrodes filled with modified ACSF (0.5–1 MOhm). Extracellular potentials were recorded with a two channel MultiClamp 700B amplifier (Axon Instruments, Molecular Devices, USA), filtered at 5 KHz and digitized at 20 KHz with a Digidata 1200B (Axon Instruments, Molecular Devices, USA). For each slice, we obtained simultaneous recordings with two electrodes placed at different recording sites along the superficial part of layer 2/3 of the dorso-medial area of the cingulate cortex. During the recording session, the electrodes were moved to different positions (in the same hemisphere or one electrode in each hemisphere) to explore the propagation of the responses along the cortex. Electrical stimuli (square current pulses of 0.1 ms and 50–300 μA applied at 0.033 Hz) were applied to layer 1 with a concentric bipolar electrode (Frederick Haer and Co., USA). The stimulus strength was adjusted at twice the threshold value. Stimulation, acquisition, and off-line analysis were done with pClamp 9.2 (Axon Instruments, Molecular Devices, USA). The position of the slices along the rostro-caudal axis is indicated by the distance to the bregma point; this distance was taken from the mouse brain atlas by Paxinos and Franklin [48] after identifying the slice with the help of anatomical marks. We considered ACC slices those placed between 1.34 and 0.38 mm from the bregma, and considered RSC slices those placed between -1.06 and -2.06 mm from the bregma (see Results, below).

Bicuculline, serotonin, (±)-2,5-Dimethoxy-4-iodoamphetamine hydrochloride (DOI) and 8-hydroxy-2-2(di-*n*-propylamino)tetralin (8-OH-DPAT) were obtained from Sigma-Aldrich (USA) and CP93129 was obtained from Tocris (UK). All drugs were applied to the slices dissolved in the extracellular medium at the indicated concentrations. All drugs were used from stock solutions prepared before the experiment; the stock solution of bicuculline was 10 mM in water; all other drugs were used from stocks prepared in water at a concentration 1000 times the final concentration used in the experiments. Data are given as mean ± SEM and the number of cases; statistical comparisons were made with SigmaStat 3.2 (Systat Software Inc., USA). Student’s t tests for unpaired and paired samples were used after checking for the normality and equal variance of the samples; when the criteria of normality and / or equal variance were not met, we used the Mann-Whitney rank sum test or the Wilcoxon signed rank test, respectively.

## Results

### Electrophysiological responses recorded in the presence of GABA receptor blockers

**Fig 1:**
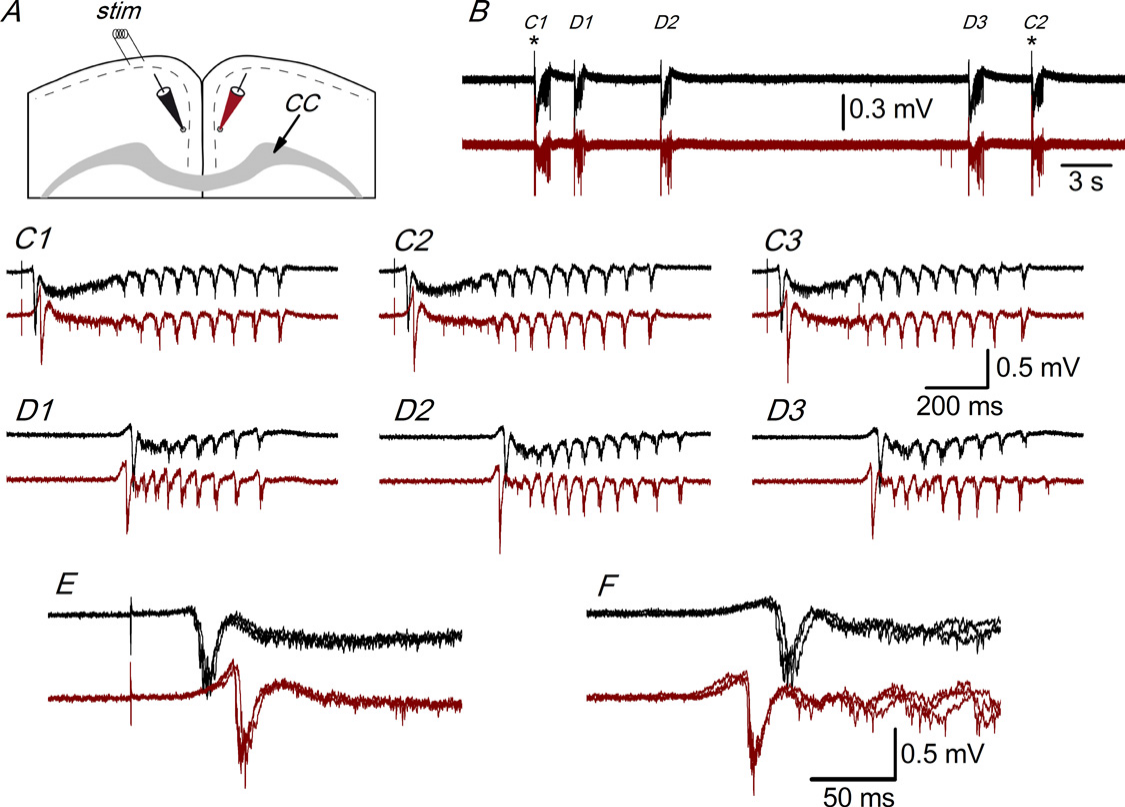
Interhemispheric propagation of synchronous discharges. A, Drawing of a coronal slice indicating the position of the stimulus electrode ("*stim*") and two extracellular recording electrodes. CC: corpus callosum. B, Simultaneous extracellular recordings obtained with electrodes placed as shown in panel A; black trace: recording from the ipsilateral hemisphere (with respect to the stimulus electrode); dark red trace: recording from the contralateral hemisphere. In both recordings, there are evoked responses (C1 and C2) and spontaneous responses (D1-D3); asterisks mark the stimulation time. C1-C3, Evoked responses from the recordings of panel B shown at enlarged scale; C3 is the following evoked response not shown in panel B. D1-D3, Spontaneous responses from panel B shown at enlarged scale. The initial negative spikes of the evoked (C1-C3) and spontaneous (D1-D3) responses are shown enlarged and superimposed in panels E and F, respectively. Recordings from a P17 animal.

We have studied the electrophysiological responses recorded with extracellular electrodes in the superficial part of layer 2/3 of coronal slices prepared from juvenile mice (14 to 20 postnatal days; P14 to P20) bathed in modified ACSF (with 5 mM KCl, 1.2 mM CaCl_2_ and 1 mM Mg^++^; see Methods). Fig 1 shows the arrangement of the stimulating and recording electrodes in the dorso-medial part of the cingulate cortex in coronal slices in which a large part of the fibers forming the corpus callosum remained intact. In the presence of 10 μM bicuculline (a GABA_A_ receptor blocker) we recorded large all-or-nothing discharges that appeared spontaneously or that were evoked by layer 1 stimulation (Fig 1). These extracellular responses were caused by the synchronous firing of neurons that takes place in the presence of bicuculline (or other GABA_A_ receptor blockers [19, 20, 21]. The size, shape, and time course of the synchronous discharges recorded in a particular slice did not change throughout the recording time (that in some cases was as long as 2 hours) and were very similar for either evoked or spontaneous discharges (Fig 1). The synchronous discharges evoked by stimulation of layer 1 in the presence of 10 μM bicuculline propagated from the stimulation site along the superficial part of layer 2/3; they also propagated to layer 2/3 of the contralateral cortex (relative to the stimulated side) through the corpus callosum (Fig 1). The synchronous responses that appeared spontaneously also propagated within a hemisphere and to the contralateral hemisphere; however, while the evoked responses always propagated from the ipsilateral to the contralateral hemisphere (as expected, because the evoked responses were generated close to the stimulus electrode), the spontaneous responses could have originated in either hemisphere and propagated to the opposite side. In the example illustrated in Fig 1, the spontaneous responses appeared first in the contralateral (relative to the stimulus electrode) hemisphere and they propagated to the ipsilateral side (Fig 1F), in contrast to the evoked responses (Fig 1E). In some slices, the hemisphere in which the spontaneous discharges originated changed several times during prolonged recordings; however, independently of the site of origin of the spontaneous discharges, their size and shape did not change, and was always similar to the evoked responses recorded in the same slice.

### Differences between the ACC and RSC in the intra- and interhemispheric propagation of synchronous discharges

**Fig 2:**
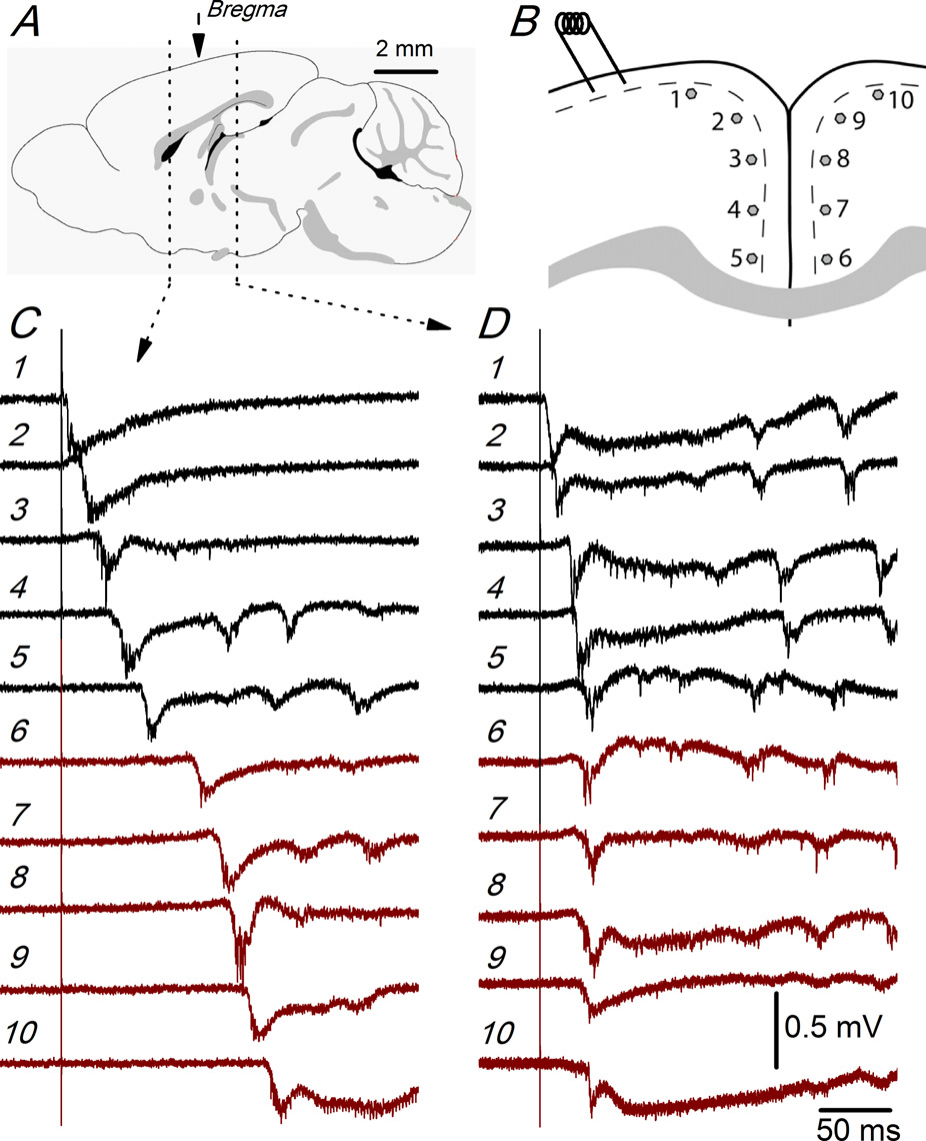
Propagation of synchronous discharges in the ACC and RSC. A, Sagital drawing of the mouse brain showing the two rostro-caudal levels from which the recordings shown in panels C and D were obtained. The middle arrow shows the position of the bregma reference point. The rostral level to the bregma is at the ACC and the caudal level to the bregma is at the RSC. Fig taken from the mouse brain atlas of the Allen Institute (www.brain-map.org). B, Position of the stimulus electrode and the 10 recording sites used in these experiments; in ACC slices, the average distance between recording sites was 440 μm; in RSC slices, the relative position of the recording sites (in both hemispheres) was the same, but the average distance between recording sites was 370 μm. C and D, Recordings obtained at each of the recording sites shown in panel B obtained from an ACC slice (C; 0.98 mm from the bregma) and a RSC slice (D; -1.06 mm from bregma); the numbers next to the recordings correspond to the recording site numbers in B. Recordings shown in panels C and D are from two slices of the same P19 mouse.

Fig 2 illustrates the propagation of evoked discharges within the ipsilateral (relative to the stimulated side) and to the contralateral hemispheres of the ACC and RSC. To explore this propagation, we used simultaneous recordings obtained with two extracellular electrodes that were moved along 10 predetermined recording sites (recording sites #1 to #5 in the ipsilateral hemisphere and #6 to #10 in the contralateral hemisphere) placed at equal intervals in the superficial part of layer 2/3 of the dorso-medial cortex of both hemispheres; the recording sites were separated, on average, by 440 μm in ACC slices and by 370 μm in RSC slices (Fig 2B). The propagation of the evoked discharges is indicated by the increase in the latency of the responses recorded at different recording sites (Fig 2C and 2D). The latencies of the responses of the example recordings shown in Fig 2 clearly show that this propagation was different in rostral (ACC) compared to caudal (RSC) slices.

**Fig 3:**
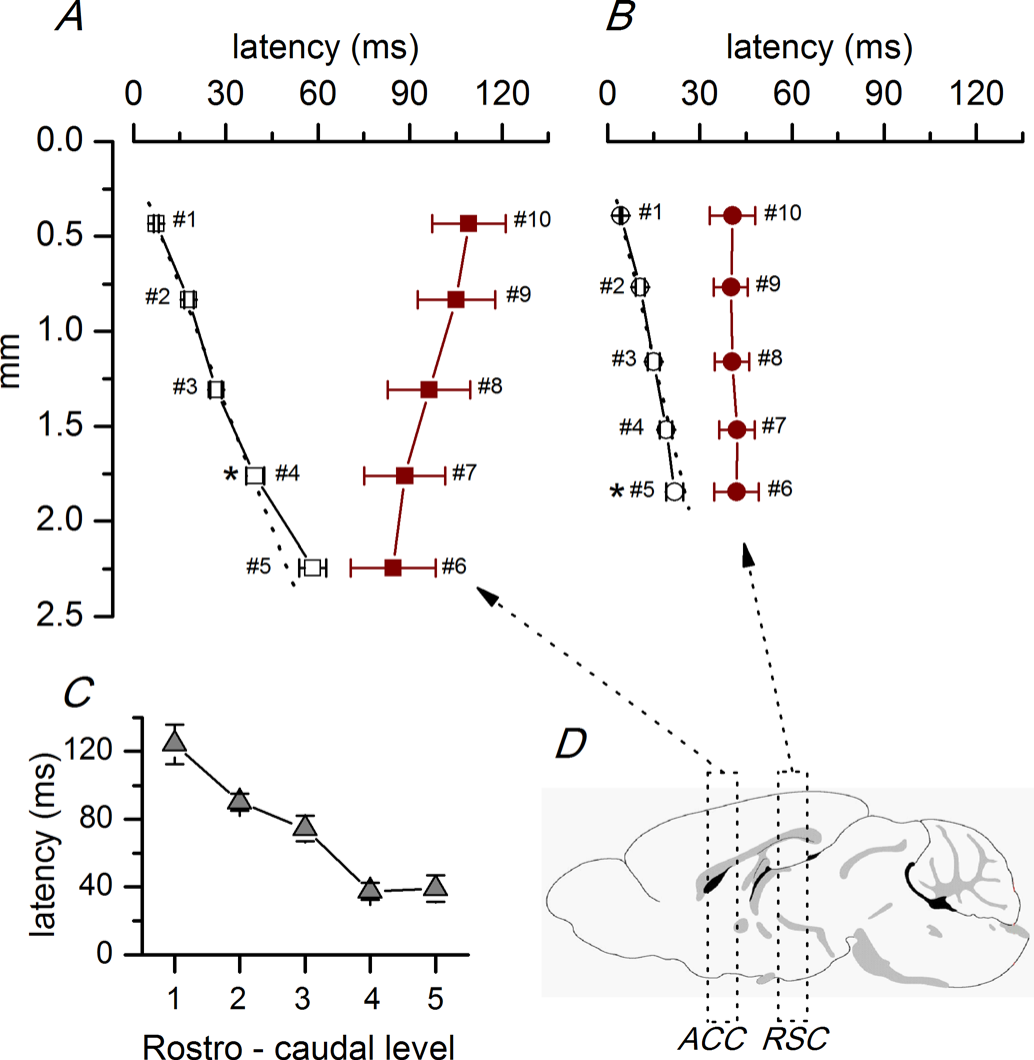
Latencies of synchronous discharges propagated along the ACC and RSC. A, Plot of the average latencies of the discharges recorded at different recording sites in the anterior cingulate cortex (n = 7 slices located between 1.34 and 0.38 mm from the bregma) in the ipsilateral (white symbols) and contralateral (dark red symbols) hemispheres. B, Plot of the average latencies of the discharges recorded at different recording sites in the retrosplenial cortex (n = 5 slices located between -1.06 and -2.06 mm from the bregma) in the ipsilateral and contralateral hemispheres (white and dark red symbols, respectively). The numbers next to each data point are the recording site number as described in Fig 2B). The distance of each recording site was measured with respect to the stimulus electrode in the ipsilateral cortex; in the contralateral cortex, the recording sites were placed symmetrically with respect to the ipsilateral side (see Fig 2B). The dotted lines show the linear fit to the latencies and the propagation velocities were calculated from the slope of these fits; the linear fit was done to the three dorsal values (recording sites #1-#3 as in Fig 2B). The difference of the latencies measured at a similar distance from the stimulus electrode (~1800 μm; recording site #4 in the ACC and #5 in the RSC) in ACC and RSC slices was statistically significant (* p<0.05 Student’s t test). C, Averaged latencies of the discharges recorded at the same position in the contralateral cortex (~400 μm from the most ventral recording site; recording site #7 as in Fig 2B) at different rostro–caudal levels, including the ACC and RSC. The level of the slices respect to the bregma shown in the horizontal axis were (in mm): 1, 1.34/0.86; 2, 0.62/0.14; 3, -0.10/-0.58; 4, -0.82/-1.34; 5, -1.58/-1.82. D, Sagital drawing of the mouse brain (same drawing as Fig 2A) showing the grouping of slices into ACC and RSC levels described in the text. The data points used to calculate the averages in panels A, B and C are available in S1 Table.

**Fig 4:**
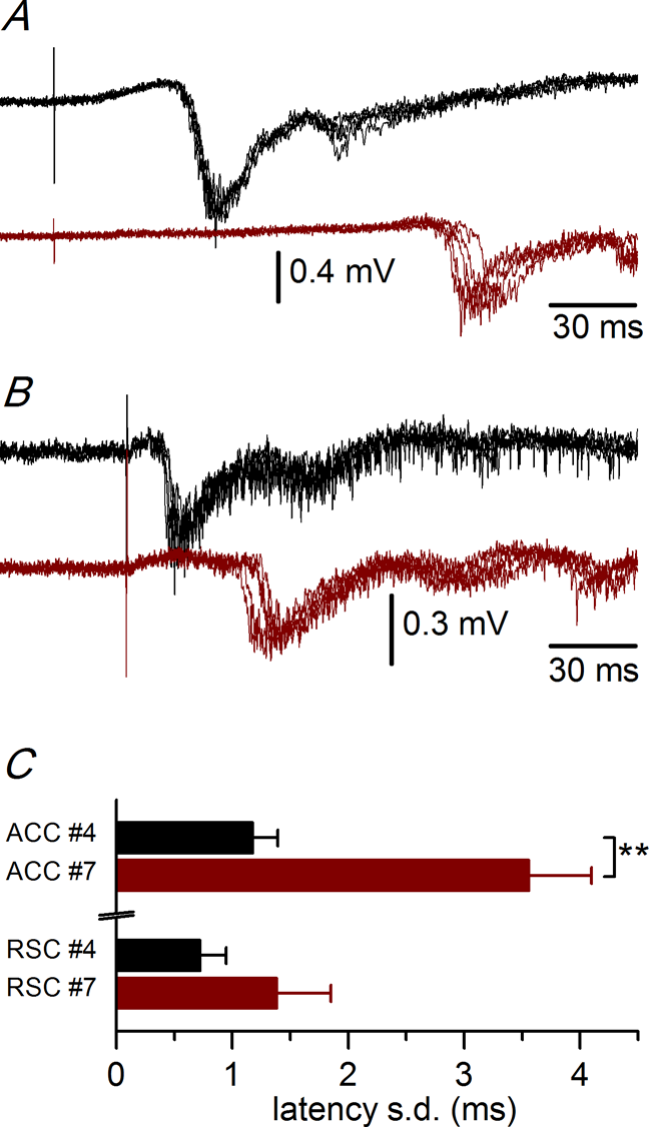
Latency variability in the ACC and RSC. A, Example of latency variability in the recordings from an ACC slice (P19 animal, 0.62 mm from the bregma); black traces: ipsilateral recordings (at recording site #4) and dark red traces: contralateral recordings (at recording site #7). B, Example of latency variability from a RSC slice (P16 animal, -1.06 mm from the bregma); black trace: ipsilateral recordings (at recording site #4) and dark red traces: contralateral recordings (at recording site #7). Five consecutive responses superimposed in panels A and B. C, Average values of the standard deviations of the latencies obtained from series of 10 consecutive responses** p< 0.01 Mann-Whitney Rank Sum test. ACC ipsilateral (recording site #4) n = 55; ACC contralateral (recording site #7) n = 27; RSC ipsilateral (recording site #4) n = 7; RSC contralateral (recording site #7) n = 5. The data points used to calculate the averages in panel C are available in S2 Table.

To study in detail the intra- and interhemispheric propagation of synchronous responses, we analyzed (Fig 3 and S1 Table) ACC and RSC slices separately. In ACC slices (Fig 3A), the discharges propagated along the superficial layer 2/3 in a dorso-ventral direction starting at the stimulus electrode at a uniform propagation velocity of 43.0 mm/s (calculated from the linear fit to the latencies of the discharges recorded at sites #1 to #3). In the most ventral part of the ACC, the propagation velocity was lower (25.9 mm/s, calculated from the slope of the latencies of the responses recorded at sites #4 and #5). After an interval of about 30–40 ms, the discharges appeared in the ventral part of the contralateral cortex and propagated in a ventro-dorsal direction at a velocity of 62.9 mm/s (calculated from the linear fit to the latencies of the discharges recorded at sites #6 to #10; Fig 3A). In RSC slices, the propagation was different (Fig 3B); in the ipsilateral side, the discharges propagated in a dorso-ventral direction as in rostral slices, but at a higher velocity (72.9 mm/s, calculated from the linear fit to the latencies of the discharges recorded at sites #1 to #3). In the most ventral part of the RSC, the propagation velocity was higher (117.0 mm/s, calculated from the slope between the latencies at recording sites #4 and #5). The latency of the responses recorded at the same distance from the stimulus electrode (about 1.8 mm; recording site #4 in rostral slices and #5 in caudal slices) was significantly (p<0.05; Student’s t test) higher in ACC than in RSC slices (Fig 3, asterisks; ACC: 39.53 ± 2.83 ms, n = 7; RSC: 21.99 ± 2.75 ms, n = 5). In the contralateral hemisphere of RSC slices, the discharges appeared after a shorter interval (about 20 ms) than in ACC slices, and almost simultaneously in the whole cortical area explored (Fig 3B). These changes in propagation seem to appear progressively in a rostro-caudal direction in the ACC cortex, as illustrated by the change in the latency of the responses measured at the same distance from the stimulus electrode in the whole rostro-caudal range (Fig 3C). A further indication of the differences in propagation between the ACC and RSC slices was the variability of the latency (latency jitter, Fig 4 and S2 Table). The latency jitter of the discharges was always higher in the contralateral hemisphere, but was also significantly higher in ACC than in RSC slices.

### Modulation by serotonin of the intra- and interhemispheric propagation of synchronous discharges in the ACC

**Fig 5:**
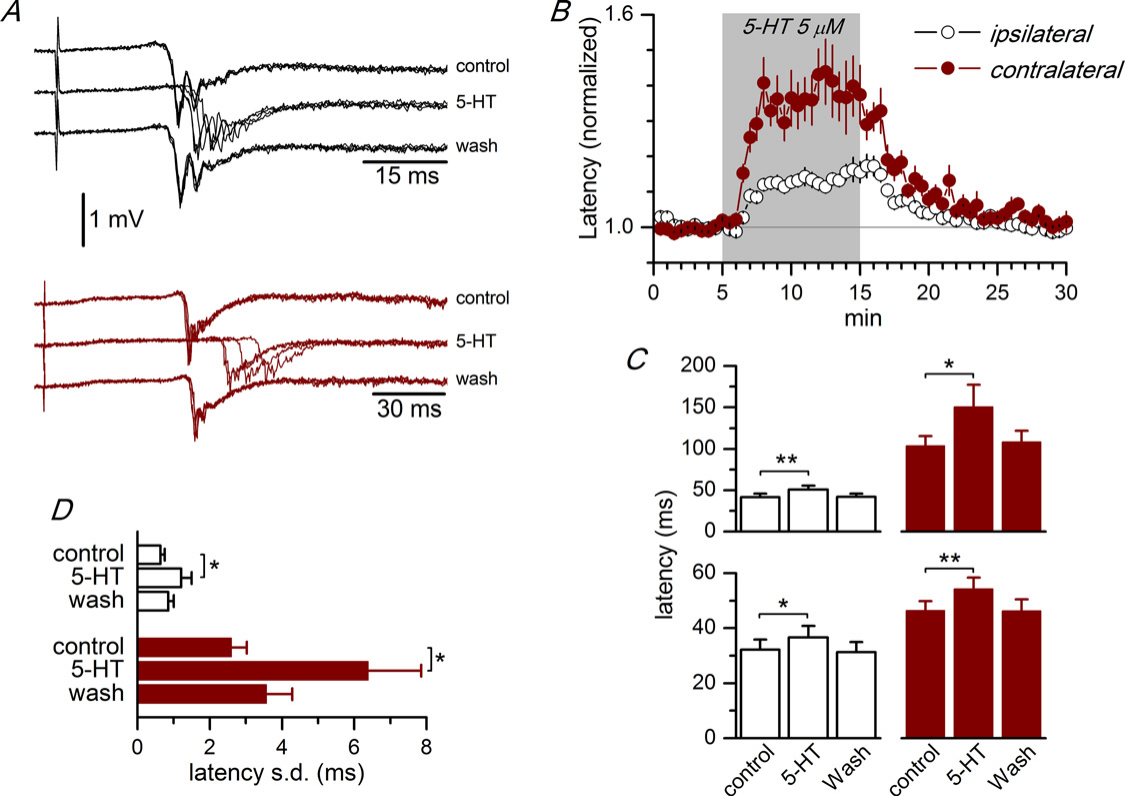
Effect of 5-HT on the propagation of synchronous discharges. A, Effect of the application of 5 μM 5-HT on the latency of the synchronous discharges recorded from an ipsilateral recording site (black traces, recording site #4; approximately at 1760 μm from the stimulus electrode) and from a contralateral recording site (dark red traces, recording site #7) in an ACC slice (P17 animal, -0.34 mm from the bregma); four consecutive responses superimposed in each trace. B, Time course of the effect of 5-HT on the latency of the synchronous discharges in ACC slices. 5-HT (5 μM) was applied during the time marked by shadow area. The plot shows the average of the values of the latency of the responses obtained from ipsilateral (white symbols, n = 32) and contralateral (dark red symbols, n = 22) recording sites; the latencies were normalized with respect to the average value of the control period. C, Absolute values of the latency of synchronous discharges obtained from ACC slices (upper histograms; n = 8 ipsilateral, 5 contralateral) and RSC slices (lower histograms; n = 3 ipsilateral, 4 contralateral) at recording sites #4 (ipsilateral, white columns) and #7 (contralateral, dark red columns). *p<0.05, **p<0.01, Student’s t test for paired samples. D, Variability of the latency of the evoked synchronous discharges measured as the standard deviation of the latencies of a series of 10 consecutive responses in ACC slices. White bars, values from recordings at recording site #4 (n = 6); dark red bars, values from recordings at recording site #7 (n = 5). * p<0.05, Student’s t test for paired samples. The data points used to calculate the averages in panels C and D are available in S3 Table.

We explored the effect of serotonin on the propagation of synchronous discharges in the ACC. The application of 5 μM serotonin had a clear effect on the propagation of the synchronous responses along the superficial part of layer 2/3 and also on the initiation of this type of activity in the cortex of the contralateral hemisphere. Serotonin increased the latency of the evoked synchronous discharges at all recording sites tested, in both the ipsilateral and contralateral hemispheres (Fig 5 and S3 Table). In the ipsilateral hemisphere, the application of 5 μM serotonin induced an 18.9% increase in the latency; this increase was steady during the application of serotonin and fully reversed after the washout of the drug (Fig 5A–5C). The latency increase induced by 5-HT was larger in the contralateral than in the ipsilateral hemisphere (40.2% and 18.9% increase, respectively). The application of 1 μM serotonin induced a lower but yet significant latency increase: 8.0% in the ipsilateral hemisphere (recording sites #4 and #5: 43.15 ± 3.25 ms control, 46.59 ± 2.90 ms 5-HT; n = 8 p<0.05; Student’s t test for paired samples; see S4 Table) and 9.1% in the contralateral hemisphere (recording site #7: 74.30 ± 17.92 ms control, 81.03 ± 18.15 ms 5-HT; n = 6 p<0.05; Student’s t test for paired samples; see S1 Table). We tested the effect of 5 μM 5-HT in the RSC and we found a similar increase in the latency of the evoked discharges in both hemispheres (Fig 5C, lower panel). In parallel with the latency increase, 5-HT also induced a large increase in the latency variability (Fig 5D), particularly in the contralateral side.

**Fig 6:**
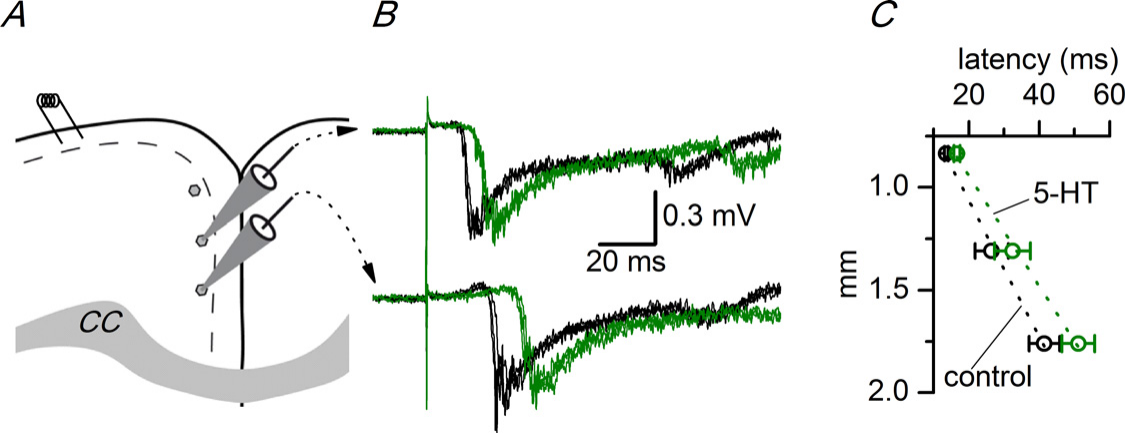
Effect of 5-HT on the propagation velocity of evoked discharges. A, Drawing of a coronal slice of the ACC showing the stimulus electrode and the three recording positions used to calculate the propagation velocity along layer 2/3. The recording sites were separated by 0.45 mm. CC: corpus callosum. B, Two simultaneous recordings obtained from the two more ventral recording sites shown in A. Three consecutive responses from the control period (black traces) and in the presence of 5 μM 5-HT (green traces). C, Change in the propagation velocity induced by 5 μM 5-HT in the ipsilateral cortex; the conduction velocity was calculated as the slope of the linear fit (dotted lines) to the latencies measured at the three recording sites shown in panel A. Control: 33.92 mm/s, 5-HT: 27.43 mm/s (n = 2–8). The vertical axis shows the distance measured from the stimulus electrode. The data points used to calculate the averages in panel C are available in S5 Table.

**Fig 7:**
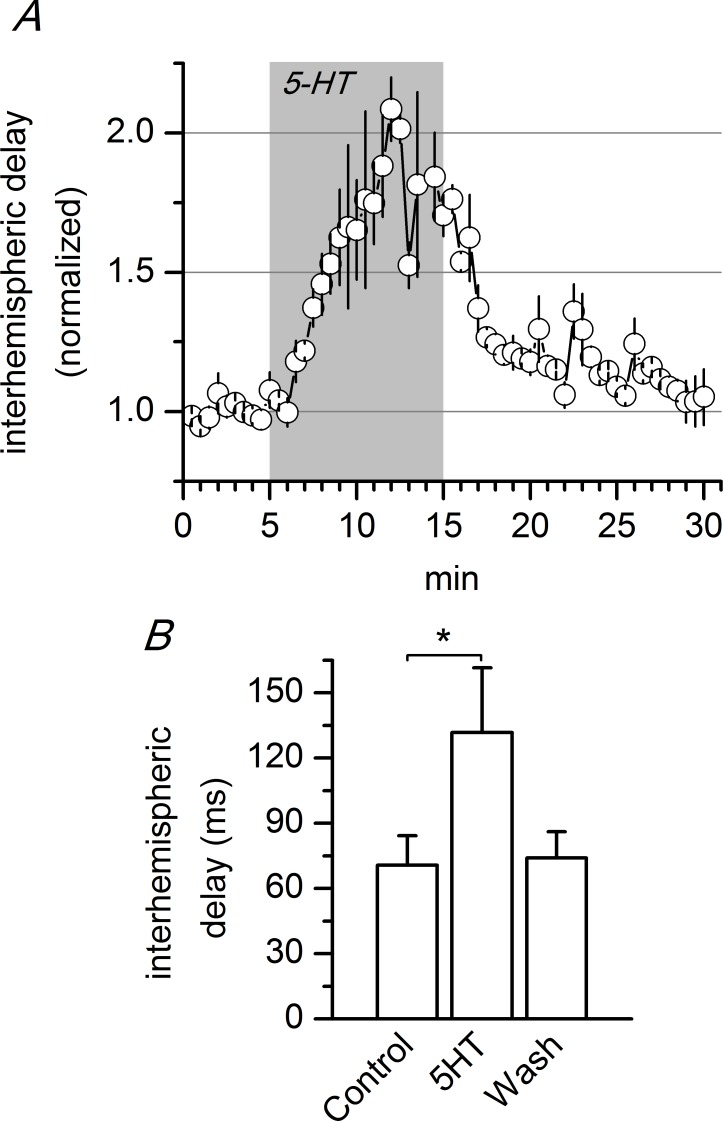
Effect of 5-HT on the interhemispheric propagation delay in the ACC. A, Time course of the interhemispheric delay and effect of 5 μM 5-HT (n = 3). B, Absolute values of the interhemispheric delay measured during the last 3 minutes of control, the last 3 minutes of application of 5-HT, and the last 3 minutes of the washout of 5-HT (n = 3); the interhemispheric delay was calculated as the difference in latency between the recordings obtained at recording sites #4 (ipsilateral) and #7 (contralateral; see Fig 5B). * p<0.05, Student’s t test for paired samples. The data points used to calculate the averages in panel B are available in S6 Table.

The latency increase induced by 5-HT in the ipsilateral side was the consequence of a decrease in the propagation velocity of synchronous responses caused by this neurotransmitter. Fig 6 shows the change in the propagation velocity of synchronous discharges induced by 5-HT in the ACC; the propagation velocity was measured from the slope of the linear fit to the latencies measured in three recording sites (#2, #3 and #4; see S5 Table) in control and in the presence of 5 μM 5-HT. This neurotransmitter caused a decrease of 19% in the propagation velocity. Serotonin also caused an increase in the time required to initiate the synchronous discharges in the contralateral cortex (interhemispheric propagation time) in the AAC; Fig 7 shows the interhemispheric propagation time measured as the difference in latency between simultaneous recordings obtained from symmetric recording sites (recording sites #4 and #7 as depicted in Fig 2B; see also S6 Table). The application of 5 μM serotonin induced a large (~180%) reversible increase in this time interval, indicating that 5-HT increased the time necessary for the propagation of the synchronous discharges to the contralateral side.

**Fig 8:**
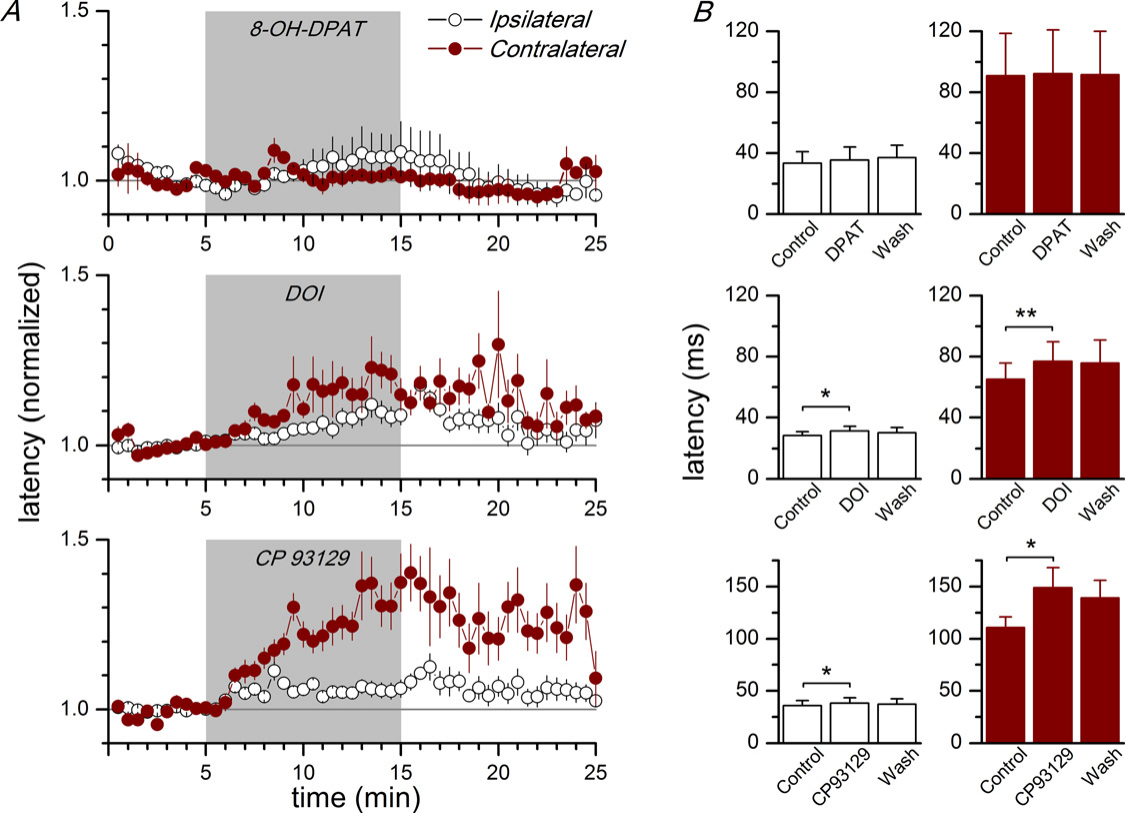
Effect of agonists of 5-HT receptors on the latency of synchronous discharges. A, Time course of the latency of synchronous discharges evoked by stimulation of layer 1 during the application of 10 μM 8-OH-DPAT (5-HT_1A_ agonist, upper panel; ipsilateral and contralateral n = 4), 10 μM DOI (5-HT_2A_ agonist, middle panel; ipsilateral n = 22, contralateral n = 10), and 10 μM CP93129 (5-HT_1B_ agonist, bottom panel; ipsilateral n = 12, contralateral n = 6). The agonists were applied during the time marked by the gray area; latencies are given as percentage of the control value (white symbols are ipsilateral latencies and dark red symbols are contralateral latencies). B, Effect of the application of 10 μM 8-OH-DPAT (upper histograms; ipsi- and contralateral n = 4), 10 μM DOI (middle histograms; ipsilateral n = 22, contralateral n = 10) and 10 μM CP93129 (lower histograms; ipsilateral n = 12, contralateral n = 6) on the latencies of synchronous discharges evoked by stimulation of layer 1 and recorded at ipsilateral recording sites (white columns) and at contralateral recording sites (dark red columns). In each plot, there are pooled values from several recording sites. Latencies were measured and averaged during the last 5 minutes of control, the last 5 minutes of drug application, and the last 5 minutes of the washout. * p<0.05; **p<0.01 with respect to the control (Student's t test for paired samples). The data points used to calculate the averages in panel B are available in S7 Table.

To investigate the subtypes of serotonin receptors implicated in the latency increase induced by 5-HT, we tested the effect of agents acting on the 5-HT receptor subtypes 5-HT_1A_, 5-HT_1B_, and 5-HT_2A_, which are present in the neocortex [35, 49]. The application of agonists of the 5-HT_1B_ or 5-HT_2A_ subtypes (CP93129 and DOI, respectively, applied at 10 μM) induced a reversible increase in the latency that mimicked the effect of 5 μM 5-HT (Fig 8 and S7 Table); however, the effect of each one of these agonists was smaller than the effect of 5 μM 5-HT: in the ipsilateral side, CP93129 induced a 6.0% increase in latency and DOI induced a 10.3% increase, while 5 μM 5-HT induced an 18.9% increase in the latency of evoked discharges. In the contralateral side, CP93129 and DOI induced a 34.4% and an 18.2% increase in latency, percentages lower than the 40.2% induced by 5-HT itself. In contrast, the 5-HT_1A_ subtype does not appear to be implicated in the modulation of the propagation (intra and interhemispheric) of synchronous discharges: the application of 10 μM DPAT (a 5-HT_1A_ agonist) did not have a significant effect on the latency of the responses in the ipsi- or contralateral hemispheres (Fig 8). These data shows the implication of the 5-HT_1B_ and 5-HT_2A_ types of serotonin receptors in the modulation of the propagation of epileptiform activity in the cingulate cortex.

## Discussion

In the present work, we have studied the properties and propagation of synchronous discharges recorded in the superficial part of layer 2/3 of the cingulate cortex in response to layer 1 stimulation; these discharges were evoked in the presence of 10 μM bicuculline. Our results show that the stimulation of layer 1 evoked, in an all-or-nothing manner, discharges formed by a negative spike followed by a slow negativity similar to the interictal or epileptiform discharges described in the neocortex after the block of GABA receptors [21, 22, 23, 50]. These discharges propagated along the superficial part in the layer 2/3 of the cingulate cortex and to the contralateral cortex through the corpus callosum; our results show important differences between the anterior cingulate cortex and the retrosplenial cortex in this intra- and interhemispheric propagation. In addition, we show that this intra- and interhemispheric propagation is strongly modulated by serotonin and that serotonin receptor subtypes 5-HT_1B_, and 5-HT_2A_, but not 5-HT_1A_ are implicated in this modulatory effect.

### Intra- and interhemispheric propagation: differences between the ACC and RSC

The synchronous discharges propagated from the initiation site (close to the stimulus electrode) along the superficial part of layer 2/3 in a dorsal to ventral direction at a uniform velocity, with the exception of the more ventral part of the cingulate cortex, where the propagation velocity was different from the rest of the studied cortex. Given that in the slices used in our experiments a number of axons connecting both hemispheres through the corpus callosum remained intact, these synchronous discharges also reached the contralateral hemisphere, in accordance with [24, 25, 51]. We studied the intra- and interhemispheric propagation of synchronous discharges in slices of the anterior cingulate cortex (slices located between 1.34 and 0.38 mm from the bregma) and the retrosplenial cortex (slices located between -1.06 and -2.06 mm from the bregma), and we found clear differences between these cortical levels. The propagation velocity in the ipsilateral hemisphere was slower in ACC slices (43.0 mm/s) compared to RSC slices (72.9 mm/s); also, in the ventral part of the ipsilateral hemisphere, the propagation velocity (calculated from the slope of the latencies at recording sites #4 and #5 of Fig 6) was different: while in ACC slices the propagation slowed down (to 25.9 mm/s), in RSC slices it accelerated (up to 117.0 mm/s). These propagation velocities were within the same range as the propagation velocities described previously for epileptiform activity or interictal discharges induced by the block of GABAergic receptors [24], but were much faster than the propagation of epileptiform activity induced by bathing the slices in zero Mg^2+^ extracellular solution [52, 53], a condition in which the feed-forward inhibition remains intact. The different propagation velocity in the ACC and RSC is probably a consequence of the different structure of the neuronal microcircuits of layer 2/3 in both cortical areas. A clear difference in the structure of layer 2/3 of the ACC and RSC cortices is the presence of a dense granular layer below layer 1 in the RSC [2], which is characterized by the presence of pyramidal neurons with a late-spiking firing pattern [7] and a characteristic mini-columnar structure of the apical dendrites of pyramidal neurons reaching layer 1 (reviewed in [6]). Since the propagation of epileptiform discharges evoked in the presence of bicuculline is heavily dependent on horizontal excitatory connections [23], the higher propagation velocity found in the RSC predicts stronger horizontal excitatory connections, longer horizontal connections, or a combination of both in the RSC, compared to the ACC.

Another difference between ACC and RSC slices was the interhemispheric delay (the difference in latency between symmetric recording sites), which was longer in ACC than in RSC slices. In our experiments, it is difficult to interpret the interhemispheric delay since it was not possible to know exactly where the synchronous discharges are initiated in the contralateral hemisphere in a particular slice. However, the difference in interhemispheric time must be related to the mechanism of initiation of the discharges in the contralateral side since it was much longer than the callosal axonal conduction time, which is close to 8 ms in the mouse [54]. A further observation that reinforces the hypothesis that the difference in the interhemispheric delay between ACC and RSC slices is due to differences in the process of generation of the discharges is the larger variability in the latency in the contralateral side in ACC slices with respect to RSC slices. In the contralateral hemisphere of the RSC the onset of the discharges was almost simultaneous in the cortical area explored. We cannot exclude the possibility that antidromic activation of contralateral layer 2 neurons by the electrical stimulus could contribute to an earlier initiation of discharges in the dorsal cortex; also, the higher propagation velocity in the RSC probably is caused by longer and / or more densely connected horizontal collateral, which also could contribute to a faster and almost simultaneous initiation of discharges in the contralateral side.

### Modulation by 5-HT and receptor agonists

The application of 5-HT increased the latency of evoked synchronous discharges. This increase was related linearly to the distance of the recording electrode with respect to the stimulation site and, therefore, it means that 5-HT decreased the propagation velocity of synchronous discharges along the superficial part of layer 2/3. In addition, 5-HT increased the latency of the discharges in the contralateral cortex, increasing the time necessary for the initiation of discharges in the contralateral side. Our results suggest a crucial role of 5-HT on the propagation of synchronous discharges evoked in disinhibited slices, in concordance with other reports showing the inhibitory actions of 5-HT on cortical network activity, such as the inhibition by 5-HT of low-Mg^++^ induced epileptiform activity in the entorhinal cortex [55].

The propagation of synchronous discharges (and its velocity) depends on feed-forward excitation mediated by AMPA receptors [23]; our results show the participation of 5-HT_2A_ and 5-HT_1B_ receptors in this action, but the lack of effect of 5-HT_1A_ receptors. This absence of effect of 5-HT_1A_ receptors is similar to the findings in other report [24], which shows no effect of 8-OH-DPAT (an agonist of 5-HT_1A_ receptors) on the pattern and propagation of epileptiform activity recorded in slices from mouse neocortex bathed with bicuculline. The effect of 5-HT decreasing the propagation velocity could be explained by at least two different mechanisms, which are triggered by the activation of 5-HT receptors. First, the direct inhibition of EPSPs at the presynaptic level, which should contribute to decrease the propagation velocity; this effect has been described in the cerebral cortex, and it is associated to the activation of 5-HT_1B_ receptors [39, 42], as well as 5-HT_1A_ and 5-HT_2A_ receptor subtypes [43, 56]. Second, the hyperpolarization of cortical pyramidal neurons, which should make them less excitable and, therefore, less prone to reaching the threshold in response to incoming EPSPs; this hyperpolarization has been described in the cortex and is an effect mediated by activation of the 5-HT_1A_ receptors in layer 5 pyramidal neurons [57, 58] and in layer 2/3 pyramidal neurons [59]. Since 5-HT_1A_ receptors do not seem to participate in the inhibition of the propagation of synchronous discharges in the cingulate cortex, this effect should be due to the presynaptic inhibition of glutamatergic EPSPs by 5-HT_1B_ and 5-HT_2A_ receptors. In addition to these two mechanisms, the activation of cortical GABAergic neurons by 5-HT_2A_ and 5-HT_1A_ receptors has been shown [60, 61, 62], which could enhance the inhibition exerted by these neurons on pyramidal neurons; however, this mechanism should not play a significant role in the inhibition of the propagation of synchronous discharges that we describe since the level of inhibition does not seem to control the propagation velocity of synchronous discharges [23], although it exerts a powerful control on network-dependent cortical activity [63].

## Supporting Information

S1 Table The Table gives the values of the latencies (in ms) of the synchronous responses recorded at different recording sites in anterior cingulate cortex (ACC) and restrosplenial cortex (RSC). **S1A Table**; latencies of the responses recorded at the ACC (n = 7 slices). These values are averaged and shown in Fig 3A of the main text. **S1B Table**; latencies of the responses recorded at the RSC (n = 5 slices). These values are averaged and shown in Fig 3B of the main text. **S1C Table**; latencies recorded at recording site #7 in slices from different rostro–caudal levels. The rostro-caudal levels are defined by the distance (in mm) respect to the bregma reference point, being positive values rostral to bregma and negative values caudal to bregma (n = 2–16 slices). These values are averaged and shown in Fig 3C of the main text
(PDF)

S2 Table Values (in ms) of the standard deviation (S.D.) of the latencies recorded in the anterior cingulate cortex (ACC) and retrosplenial cortex (RSC) at an ipsilateral recording site (recording site #4) and a contralateral recording site (recording site #7). Each value is the standard deviation of the latencies of ten consecutive responses recorded in one slice at the indicated recording site. The values in this table are averaged in Fig 4C of the man text.
(PDF)

S3 Table Effect of the application of 5 μM 5-HT on the latencies of the synchronous discharges recorded in the anterior cingulate cortex (ACC) and the retrosplenial cortex (RSC). **S3A Table**, Effect of the application of 5 μM 5-HT in Anterior Cingulate Cortex slices (values averaged and shown in Fig 5C, upper panel, of the man text). Data in ms; n = 8 slices (ipsilateral), n = 5 slices (contralateral). **S3B Table**; Effect of the application of 5 μM 5-HT in Retrosplenial Cortex slices ((values averaged and shown in Fig 5C, lower panel, of the main text). Data in ms; n = 3 slices (ipsilateral), n = 4 slices (contralateral). **S3C Table**; Effect of the application of 5 μM 5-HT on the standard deviation (S.D.) of the synchronous responses recorded in Anterior Cingulate Cortex slices (values averaged and shown in Fig 5D of the main text). Data in S3C Table are the S.D. of the latencies measured in 10 consecutive responses for each condition; n = 6 slices (ipsilateral), n = 7 slices (contralateral).
(PDF)

S4 Table Effect of the application of 1 μM 5-HT on the latencies (in ms) of the synchronous discharges recorded in the ipsilateral (S4A Table; n = 8 slices) and contralateral (S4B Table; n = 6 slices) hemispheres of the anterior cingulate cortex. Each latency values is the average of the latencies of the responses recorded in the last 3 minutes of each experimental condition. The averages of the values of this table are given in the main text.
(PDF)

S5 Table Latency data used to calculate the effect of 5-HT on the propagation velocity (main text, Fig 6C). The table gives the latencies (in ms) of the recorded responses obtained in control condition and in the presence of 5 μM 5-HT in recording sites #2 (2 slices), #3 (3 slices) and #4 (8 slices).
(PDF)

S6 Table Effect of the application of 5 μM 5-HT on the interhemispheric propagation delay. The table gives in ms the interhemispheric propagation delay measured in three slices in control conditions, during the application of 5 μM 5-HT and during the washout of the 5-HT. In each slice, the interhemispheric propagation delay was calculated as difference in the latency of the discharges recorded in recording site #4 (ipsilateral) and recording site #7 (contralateral). The data in this table are averaged and compared in Fig 7B of the main text.
(PDF)

S7 Table Effect of 5-HT receptor agonists on the latencies (in ms) of synchronous discharges. **S7A Table**; Effect of 8-OH-DPAT on Anterior Cingulate Cortex slices (ipsilateral n = 4 slices, contralateral n = 4 slices). These data are averaged and shown in Fig 8B, upper panel, of the main text. **S7B Table**; effect of DOI on Anterior Cingulate Cortex slices (ipsilateral n = 22 slices, contralateral n = 10 slices). These data are averaged and shown in Fig 8B, middle panel, of the main text. **S7C Table**; effect of CP 93129 on Anterior Cingulate Cortex slices (ipsilateral n = 12 slices, contralateral n = 6 slices). These data are averaged and shown in Fig 8B, lower panel, of the main text. In all tables: N.d. data not measured.
(PDF)
