# Supplementary material for: Intra- and Interhemispheric Propagation of Electrophysiological Synchronous Activity and Its Modulation by Serotonin in the Cingulate Cortex of Juvenile Mice
Source: PLoS One. 2016 Mar 1;11(3):e0150092. doi: 10.1371/journal.pone.0150092 (PMC4773155; doi:10.1371/journal.pone.0150092)
Supplement: S1 Table — S1A Table; latencies of the responses recorded at the ACC (n = 7 slices). These values are averaged and shown in Fig 3A of the main text. S1B Table; latencies of the responses recorded at the RSC (n = 5 slices). These values are averaged and shown in Fig 3B of the main text. S1C Table; latencies recorded at recording site #7 in slices from different rostro–caudal levels. The rostro-caudal levels are defined by the distance (in mm) respect to the bregma reference point, being positive values rostral to bregma and negative values caudal to bregma (n = 2–16 slices). These values are averaged and shown in Fig 3C of the main text (PDF) [file pone.0150092.s001.pdf]

S1 Table

S1 Table A

	**ipsilateral**					**contralateral**				
	Recording site #1	Recording site #2	Recording site #3	Recording site #4	Recording site #5	Recording site #6	Recording site #7	Recording site #8	Recording site #9	Recording site #10
Slice #1	10.21	18.45	33.84	46.68	77.20	145.28	144.3	152.40	146.50	148.16
Slice #2	6.98	19.68	24.57	32.76	41.69	49.47	56.13	62.91	72.22	80.99
Slice #3	3.71	15.06	27.91	39.83	61.07	91.95	109.12	114.18	127.04	127.06
Slice #4	4.58	13.66	22.93	28.01	37.85	38.07	37.96	46.60	57.83	68.60
Slice #5	8.05	28.55	23.55	42.40	48.58	65.90	70.81	75.83	78.17	83.45
Slice #6	10.68	21.60	30.64	42.50	54.35	78.14	88.02	96.88	118.24	107.56
Slice #7	6.21	16.33	23.53	37.58	51.86	116.60	106.40	120.90	134.24	148.20

S1 Table B

	ipsilateral					contralateral				
	Recording site #1	Recording site #2	Recording site #3	Recording site #4	Recording site #5	Recording site #6	Recording site #7	Recording site #8	Recording site #9	Recording site #10
Slice #1	5.09	11.91	14.81	22.02	32.25	34.63	36.71	39.32	41.71	44.43
Slice #2	3.72	12.01	16.09	20.35	22.93	61.08	51.13	61.85	67.62	76.31
Slice #3	5.23	8.65	11.13	17.39	18.69	34.98	33.45	31.75	35.66	34.35
Slice #4	2.76	9.06	10.77	15.96	16.98	16.85	24.63	30.28	31.02	33.99
Slice #5	4.98	11.20	15.44	18.21	20.15	62.04	61.10	51.61	46.73	33.73

S1 Table C

Latencies in slices at various rostro-caudal levels respect to bregma (in mm)									
**1.34/0.86 mm**		**0.62/0.14 mm**		**-0.10/-0.58 mm**		**-0.82/-1.34 mm**		**-1.58/-1.82 mm**	
Slice #1 (ms)	158.30	Slice #1 (ms)	70.13	Slice #1 (ms)	55.04	Slice #1 (ms)	46.88	Slice #1 (ms)	31.02
Slice #2 (ms)	104.35	Slice #2 (ms)	123.12	Slice #2 (ms)	109.66	Slice #2 (ms)	35.66	Slice #2 (ms)	46.73
Slice #3 (ms)	120.40	Slice #3 (ms)	51.96	Slice #3 (ms)	117.43	Slice #3 (ms)	29.49		
Slice #4 (ms)	95.86	Slice #4 (ms)	87.50	Slice #4 (ms)	74.63				
Slice #5 (ms)	160.84	Slice #5 (ms)	81.44	Slice #5 (ms)	91.80				
Slice #6 (ms)	106.23	Slice #6 (ms)	84.81	Slice #6 (ms)	77.02				
		Slice #7 (ms)	102.02	Slice #7 (ms)	115.86				
		Slice #8 (ms)	89.07	Slice #8 (ms)	36.83				
		Slice #9 (ms)	107.70	Slice #9 (ms)	63.25				
		Slice #10 (ms)	122.15	Slice #10 (ms)	41.71				
		Slice #11 (ms)	66.70	Slice #11 (ms)	67.62				
		Slice #12 (ms)	71.48	Slice #12 (ms)	42.69				

		Slice #13 (ms)	76.30	Slice #13 (ms)	75.64				
		Slice #14 (ms)	94.63						
		Slice #15 (ms)	99.32						
		Slice #16 (ms)	115.00						

S1 Table.

The Table gives the values of the latencies (in ms) of the synchronous responses recorded at different recording sites in anterior cingulate cortex (ACC) and restrosplenial cortex (RSC). **S1 Table A**; latencies of the responses recorded at the ACC (n = 7 slices). These values are averaged and shown in figure 3A of the main text. **S1 Table B**; latencies of the responses recorded at the RSC (n =5 slices). These values are averaged and shown in figure 3B of the main text**. S1 Table C**; latencies recorded at recording site #7 in slices from different rostro – caudal levels. The rostro-caudal levels are defined by the distance (in mm) respect to the bregma reference point, being positive values rostral to bregma and negative values caudal to bregma (n = 2-16 slices). These values are averaged and shown in figure 3C of the main text
