# Supplementary material for: Intra- and Interhemispheric Propagation of Electrophysiological Synchronous Activity and Its Modulation by Serotonin in the Cingulate Cortex of Juvenile Mice
Source: PLoS One. 2016 Mar 1;11(3):e0150092. doi: 10.1371/journal.pone.0150092 (PMC4773155; doi:10.1371/journal.pone.0150092)
Supplement: S2 Table — Each value is the standard deviation of the latencies of ten consecutive responses recorded in one slice at the indicated recording site. The values in this table are averaged in Fig 4C of the man text. (PDF) [file pone.0150092.s002.pdf]

S2 Table

**ACC** Recording site #4	**ACC** Recording site #7	**RSC** Recording site #4	**RSC** Recording site #7
1.22	1.61	1.83	0.49
0.48	2.17	0.54	1.25
0.73	3.19	0.88	0.28
1.29	1.19	0.29	2.64
0.7	1.11	1.07	2.25
1.28	3.03	0.18	--
0.45	2.89	0.28	--
0.65	1.82	--	--
1.77	3.21	--	--
3.26	1.85		
0.64	1.99		
1.3	0.79		
0.39	2.18		
1.31	4.06		
0.46	3.06		
0.58	1.56		
1.23	2.56		
2.57	1.15		
0.61	4.88		
0.49	11.68		
0.47	5.03		
1.01	10.54		
1.22	4.51		
1.91	3.38		
1.44	1.46		
0.15	7.05		
0.19	8.11		
0.6	--		
0.65			
3.14			
1.45			
2.33			
0.6			
1.49			
0.77			
1.31			
2.64			
0.39			
2.15			

0.92
1.73
0.46
0.97
2.88
0.79
2.02
0.68
0.88
1.01
0.33
2.18
0.81
1.16
1.83
0.62

S2 Table.

Values (in ms) of the standard deviation (S.D.) of the latencies recorded in the anterior cingulate cortex (**ACC**) and retrosplenial cortex (**RSC**) at an ipsilateral recording site (recording site #4) and a contralateral recording site (recording site #7). Each value is the standard deviation of the latencies of ten consecutive responses recorded in one slice at the indicated recording site. The values in this table are averaged in figure 4C of the man text.
