# Supplementary material for: Intra- and Interhemispheric Propagation of Electrophysiological Synchronous Activity and Its Modulation by Serotonin in the Cingulate Cortex of Juvenile Mice
Source: PLoS One. 2016 Mar 1;11(3):e0150092. doi: 10.1371/journal.pone.0150092 (PMC4773155; doi:10.1371/journal.pone.0150092)
Supplement: S3 Table — S3A Table, Effect of the application of 5 μM 5-HT in Anterior Cingulate Cortex slices (values averaged and shown in Fig 5C, upper panel, of the man text). Data in ms; n = 8 slices (ipsilateral), n = 5 slices (contralateral). S3B Table; Effect of the application of 5 μM 5-HT in Retrosplenial Cortex slices ((values averaged and shown in Fig 5C, lower panel, of the main text). Data in ms; n = 3 slices (ipsilateral), n = 4 slices (contralateral). S3C Table; Effect of the application of 5 μM 5-HT on the standard deviation (S.D.) of the synchronous responses recorded in Anterior Cingulate Cortex slices (values averaged and shown in Fig 5D of the main text). Data in S3C Table are the S.D. of the latencies measured in 10 consecutive responses for each condition; n = 6 slices (ipsilateral), n = 7 slices (contralateral). (PDF) [file pone.0150092.s003.pdf]

S3 Table

S3 Table A

ACC							
**Ipsilateral**				**Contralateral**			
	**control**	**5 μM 5-HT**	**washout**		**control**	**5 μM 5-HT**	**washout**
	Latency (ms)	Latency (ms)	Latency (ms)		Latency (ms)	Latency (ms)	Latency (ms)
Slice #1	62.33	71.18	63.25	Slice #1	140.62	209.77	157.25
Slice #2	47.38	56.62	49.12	Slice #2	121.60	216.85	148.8
Slice #3	43.99	56.74	42.75	Slice #3	87.50	119.08	92.43
Slice #4	47.27	62.75	51.05	Slice #4	74.03	80.79	74.35
Slice #5	22.08	33.47	23.35	Slice #5	89.07	114.10	88.15
Slice #6	42.21	49.06	42.90				
Slice #7	36.50	39.58	36.10				
Slice #8	29.64	38.84	28.25				

S3 Table B

RSC							
Ipsilateral				Contralateral			
	control	5 μM 5-HT	washout		control	5 μM 5-HT	washout
	Latency (ms)	Latency (ms)	Latency (ms)		Latency (ms)	Latency (ms)	Latency (ms)
Slice #1	26.03	29.25	29.25	Slice #1	41.71	53.08	41.95
Slice #2	33.02	40.12	40.12	Slice #2	39.10	46.88	42.12
Slice #3	35.98	42.05	42.05	Slice #3	30.73	52.66	29.75
				Slice #4	63.25	70.32	62.50

S3 Table C

ACC							
**Ipsilateral**				**Contralateral**			
	control	5 μM 5-HT	washout		control	5 μM 5-HT	washout
	Latency S.D.	Latency S.D.	Latency S.D.		Latency S.D.	Latency S.D.	Latency S.D.
	(ms)	(ms)	(ms)		(ms)	(ms)	(ms)
Slice #1	1.4	2.6	1.82	Slice #1	1.63	3.08	1.93
Slice #2	0.54	1.07	0.7	Slice #2	1.47	2.43	2.31
Slice #3	0.29	0.61	0.41	Slice #3	1.46	2.64	n.d.
Slice #4	1.07	2.01	1.17	Slice #4	4.45	12.13	6.01
Slice #5	0.18	0.46	0.36	Slice #5	4.31	13.03	5.68
Slice #6	0.28	0.46	0.4	Slice #6	1.57	3.93	2.35
				Slice #7	2.81	7.02	3.55

S3 Table.

Effect of the application of 5 μM 5-HT on the latencies of the synchronous discharges recorded in the anterior cingulate cortex (ACC) and the retrosplenial cortex (RSC). **S3 Table A**, Effect of the application of 5 μM 5-HT in Anterior Cingulate Cortex slices (values averaged and shown in figure 5C, upper panel, of the man text). Data in ms; n = 8 slices (ipsilateral), n = 5 slices (contralateral). **S3 Table B**; Effect of the application of 5 μM 5-HT in Retrosplenial Cortex slices ((values averaged and shown in figure 5C, lower panel, of the main text). Data in ms; n = 3 slices (ipsilateral), n = 4 slices (contralateral). **S3 Table C**; Effect of the application of 5 μM 5-HT on the standard deviation (S.D.) of the synchronous responses recorded in Anterior Cingulate Cortex slices ((values averaged and shown in figure 5D of the main text). Data in S3 Table C are the S.D. of the latencies measured in 10 consecutive responses for each condition; n = 6 slices (ipsilateral), n = 7 slices (contralateral).
