# Supplementary material for: Intra- and Interhemispheric Propagation of Electrophysiological Synchronous Activity and Its Modulation by Serotonin in the Cingulate Cortex of Juvenile Mice
Source: PLoS One. 2016 Mar 1;11(3):e0150092. doi: 10.1371/journal.pone.0150092 (PMC4773155; doi:10.1371/journal.pone.0150092)
Supplement: S4 Table — Each latency values is the average of the latencies of the responses recorded in the last 3 minutes of each experimental condition. The averages of the values of this table are given in the main text. (PDF) [file pone.0150092.s004.pdf]

S4 Table

S4 Table A

	Ipsilateral ACC		
	**control**	**1 μM 5-HT**	**washout**
	Latency (ms)	Latency (ms)	Latency (ms)
Slice #1	48.30	48.94	47.11
Slice #2	32.06	37.67	34.83
Slice #3	36.53	36.76	34.37
Slice #4	37.63	49.08	43.22
Slice #5	61.83	62.08	70.54
Slice #6	44.74	49.58	46.25
Slice #7	44.56	47.66	46.59
Slice #8	39.58	40.93	36.30

S4 Table B

	Contralateral ACC		
	**control**	**1 μM 5-HT**	**washout**
	Latency (ms)	Latency (ms)	Latency (ms)
Slice #1	143.72	144.56	137.17
Slice #2	68.52	81.04	75.36
Slice #3	51.92	53.44	49.70
Slice #4	42.48	46.11	45.55
Slice #5	109.73	124.48	119.73
Slice #6	29.43	36.55	29.93

S4 Table.
Effect of the application of 1 μM 5-HT on the latencies (in ms) of the synchronous discharges recorded in the ipsilateral (**S4 Table A**; n = 8 slices) and contralateral (**S4 Table B**; n = 6 slices) hemispheres of the anterior cingulate cortex. Each latency values is the average of the latencies of the responses recorded in the last 3 minutes of each experimental condition. The averages of the values of this table are given in the main text.
