# Supplementary material for: Intra- and Interhemispheric Propagation of Electrophysiological Synchronous Activity and Its Modulation by Serotonin in the Cingulate Cortex of Juvenile Mice
Source: PLoS One. 2016 Mar 1;11(3):e0150092. doi: 10.1371/journal.pone.0150092 (PMC4773155; doi:10.1371/journal.pone.0150092)
Supplement: S5 Table — The table gives the latencies (in ms) of the recorded responses obtained in control condition and in the presence of 5 μM 5-HT in recording sites #2 (2 slices), #3 (3 slices) and #4 (8 slices). (PDF) [file pone.0150092.s005.pdf]

S5 Table

Ipsilateral								
	**Recording site #2**			**Recording site #3**			**Recording site #4**	
	control	5 μM 5-HT		control	5 μM 5-HT		control	5 μM 5-HT
	Latency (ms)	Latency (ms)		Latency (ms)	Latency (ms)		Latency (ms)	Latency (ms)
Slice #1	14.19	15.73	Slice #1	31.21	37.96	Slice #1	62.33	71.18
Slice #2	20.82	22.38	Slice #2	25.18	30.68	Slice #2	46.675	56.62
			Slice #3	27.88	31.88	Slice #3	43.99	56.74
						Slice #4	32.76	39.58
						Slice #5	47.27	62.75
						Slice #6	28.13	29.36
						Slice #7	36.89	35.2
						Slice #8	46.12	54.28

S5 Table.

Latency data used to calculate the effect of 5-HT on the propagation velocity (main text, figure 6C). The table gives the latencies (in ms) of the recorded responses obtained in control condition and in the presence of 5 μM 5-HT in recording sites #2 (2 slices), #3 (3 slices) and #4 (8 slices).
