# Supplementary material for: Intra- and Interhemispheric Propagation of Electrophysiological Synchronous Activity and Its Modulation by Serotonin in the Cingulate Cortex of Juvenile Mice
Source: PLoS One. 2016 Mar 1;11(3):e0150092. doi: 10.1371/journal.pone.0150092 (PMC4773155; doi:10.1371/journal.pone.0150092)
Supplement: S6 Table — The table gives in ms the interhemispheric propagation delay measured in three slices in control conditions, during the application of 5 μM 5-HT and during the washout of the 5-HT. In each slice, the interhemispheric propagation delay was calculated as difference in the latency of the discharges recorded in recording site #4 (ipsilateral) and recording site #7 (contralateral). The data in this table are averaged and compared in Fig 7B of the main text. (PDF) [file pone.0150092.s006.pdf]

S6 Table

	control	5 μM 5-HT	washout
	(ms)	(ms)	(ms)
Slice #1	92.17	170.12	88.17
Slice #2	74.45	142.55	84.01
Slice #3	43.10	76.18	50.18

S6 Table.

Effect of the application of 5 μM 5-HT on the interhemispheric propagation delay. The table gives in ms the interhemispheric propagation delay measured in three slices in control conditions, during the application of 5 μM 5-HT and during the washout of the 5-HT. In each slice, the interhemispheric propagation delay was calculated as difference in the latency of the discharges recorded in recording site #4 (ipsilateral) and recording site #7 (contralateral). The data in this table are averaged and compared in figure 7B of the main text.
