# Supplementary material for: Intra- and Interhemispheric Propagation of Electrophysiological Synchronous Activity and Its Modulation by Serotonin in the Cingulate Cortex of Juvenile Mice
Source: PLoS One. 2016 Mar 1;11(3):e0150092. doi: 10.1371/journal.pone.0150092 (PMC4773155; doi:10.1371/journal.pone.0150092)
Supplement: S7 Table — S7A Table; Effect of 8-OH-DPAT on Anterior Cingulate Cortex slices (ipsilateral n = 4 slices, contralateral n = 4 slices). These data are averaged and shown in Fig 8B, upper panel, of the main text. S7B Table; effect of DOI on Anterior Cingulate Cortex slices (ipsilateral n = 22 slices, contralateral n = 10 slices). These data are averaged and shown in Fig 8B, middle panel, of the main text. S7C Table; effect of CP 93129 on Anterior Cingulate Cortex slices (ipsilateral n = 12 slices, contralateral n = 6 slices). These data are averaged and shown in Fig 8B, lower panel, of the main text. In all tables: N.d. data not measured. (PDF) [file pone.0150092.s007.pdf]

S7 Table

S7 Table A

8-OH-DPAT							
**Ipsilateral**				**Contralateral**			
	control	**8-OH-DPAT**	washout		control	**8-OH-DPAT**	washout
	Latency (ms)	Latency (ms)	Latency (ms)		Latency (ms)	Latency (ms)	Latency (ms)
Slice #1	43.61	41.42	42.23	Slice #1	118.67	120.80	119.92
Slice #2	21.99	21.62	25.63	Slice #2	63.11	63.68	63.36
Slice #3	48.61	57.05	57.66	Slice #3	139.22	142.95	142.38
Slice #4	19.23	21.88	23.12	Slice #4	42.85	43.73	N.d.

S7 Table B

DOI							
**Ipsilateral**				**Contralateral**			
	control	**DOI**	washout		control	**DOI**	washout
	Latency (ms)	Latency (ms)	Latency (ms)		Latency (ms)	Latency (ms)	Latency (ms)
Slice #1	37.41	49.73	57.08	Slice #1	91.8	117.34	103.39
Slice #2	11.34	12.71	13.74	Slice #2	42.69	58.24	46.13
Slice #3	34.38	29.93	29.8	Slice #3	77.02	75.57	78.56
Slice #4	30.18	42.93	33.56	Slice #4	36.83	37.74	33.64
Slice #5	41.56	45.46	47.48	Slice #5	107.70	133.70	154.88
Slice #6	14.13	14.59	15.75	Slice #6	30.13	42.88	37.61
Slice #7	35.25	32.66	30.38	Slice #7	124.35	143.42	134.20
Slice #8	19.31	17.50	16.17	Slice #8	58.21	62.44	58.48
Slice #9	28.51	26.05	27.76	Slice #9	49.63	63.30	N.d.
Slice #10	53.33	58.63	58.18	Slice #10	32.27	34.59	34.80
Slice #11	15.35	15.12	15.14				
Slice #12	15.32	16.11	15.54				
Slice #13	31.55	37.63	24.89				
Slice #14	12.88	13.93	11.08				
Slice #15	39.76	42.25	48.65				
Slice #16	19.77	24.53	22.09				
Slice #17	47.45	58.63	55.45				
Slice #18	25.03	26.18	25.19				
Slice #19	27.42	28.24	N.d.				
Slice #20	18.45	20.00	20.54				

Slice #21	42.42	49.02	42.75				
Slice #22	26.66	29.95	26.69				

S7 Table C

CP 93129							
**Ipsilateral**				**Contralateral**			
	control	**CP 93129**	washout		control	**CP 93129**	washout
	Latency (ms)	Latency (ms)	Latency (ms)		Latency (ms)	Latency (ms)	Latency (ms)
Slice #1	70.11	62.74	67.21	Slice #1	100.58	166.36	163.16
Slice #2	20.69	23.61	21.50	Slice #2	93.51	100.22	92.66
Slice #3	46.83	60.78	65.41	Slice #3	96.97	124.04	117.84
Slice #4	30.83	32.62	30.89	Slice #4	102.54	129.94	116.1
Slice #5	12.38	12.86	13.11	Slice #5	144.92	231.44	N.d.
Slice #6	59.30	68.27	59.91	Slice #6	101.14	155.56	161.22
Slice #7	16.72	18.24	17.98				
Slice #8	32.03	36.40	33.83				
Slice #9	40.75	43.11	38.76				
Slice #10	23.85	26.81	22.66				
Slice #11	35.85	37.20	39.83				
Slice #12	40.12	45.98	48.03				

S7 Table.
Effect of 5-HT receptor agonists on the latencies (in ms) of synchronous discharges. **S7 Table A**; Effect of 8-OH-DPAT on Anterior Cingulate Cortex slices (ipsilateral n = 4 slices, contralateral n = 4 slices). These data are averaged and shown in figure 8B, upper panel, of the main text. **S7 Table B**; effect of DOI on Anterior Cingulate Cortex slices (ipsilateral n = 22 slices, contralateral n = 10 slices). These data are averaged and shown in figure 8B, middle panel, of the main text. **S7 Table C**; effect of CP 93129 on Anterior Cingulate Cortex slices (ipsilateral n = 12 slices, contralateral n = 6 slices). These data are averaged and shown in figure 8B, lower panel, of the main text. In all tables: N.d. data not measured.
